# Urinary cell‐free extrachromosomal circular DNAs: A possible biomarker for chronic kidney disease

**DOI:** 10.1002/ctm2.925

**Published:** 2022-06-09

**Authors:** Selami Demirci

**Affiliations:** ^1^ Cellular and Molecular Therapeutics Branch National Heart Lung and Blood Institutes (NHLBI) National Institutes of Health (NIH) Bethesda Maryland USA

**Keywords:** cell‐free DNA, chronic kidney disease, circle‐seq, diagnosis

1

Circulating cell‐free DNA (cfDNA) floating in body fluids is an intense area of research given their potential clinical use as a class of minimally‐invasive disease biomarkers for various human diseases. While it is proposed that cfDNA is mainly the byproduct of apoptotic cells (with the major contributors being hematopoietic cells), it is also presented that constant release of nucleic acids is a sophisticated way of intercellular signalling during development, epigenetic remodelling, tissue regeneration and adjustment of the adaptive immune system.^1^ To date, cfDNA research was mostly restricted to fragmented linear DNA molecules (< 200 base pairs [bp]). Recently, however, extrachromosomal circular DNAs (eccDNAs) have gained considerable attention because of its ubiquitous prevalence in all eukaryotic cells. eccDNA is found in both physiological and pathological conditions, ranging from animals and plants to yeast sources, and have diverse origins. eccDNAs are originated from and homologous to chromosomes, but once they are formed, they are likely to act independent. Although the formation and biological roles of eccDNA remains understudied, it is thought to reflect the genome plasticity and instability.^2^ Similar to linear cf‐DNA, eccDNA is demonstrated in human plasma and considered to be released into circulation after cell apoptosis/death and turnover, with a higher rate in diseased states. Due to their covalently bonded circular structure, extracellular eccDNAs are assumed to be more resistant to exonuclease degradation compared to their linear counterparts. Considerable research has been conducted on linear cf‐DNA in blood, saliva, cerebrospinal fluid, and urine as an informative diagnostic tool^3^; however, existence of eccDNA in human urine had not been discovered. In the recent issue of “Clinical and Translational Medicine”, Lv et al. have demonstrated for the first time the presence of cell‐free eccDNA (cf‐eccDNA) in urine samples from healthy individuals and patients with advanced chronic kidney diseases (CKD).^4^ They have analyzed urine samples from 28 healthy individuals and 21 patients with advanced CKD for characterization of urinary cf‐eccDNA using a modified Circle‐Seq method.

Over one million unique urinary cf‐eccDNAs have been presented from healthy volunteer samples. As an interesting observation, the authors put forth the correlation between the number of eccDNAs and protein‐coding genes, CpG islands, short interspersed transposable elements and simple repeat elements. Most of urinary cf‐eccDNAs were noted to be smaller than 1000 bp and enriched in four main peaks positioned at 207, 358, 553 and 732 bp. While a considerable portion of the human chromosome (14.9%) contributed to the formation of urinary cf‐eccDNA, gene‐rich chromosomes including chromosome 17, 19 and 20 contributed the highest and gene‐poor chromosome 21 contributed the least. This observation and previous reports^5^, ^6^ imply a strong correlation between transcriptional activity and eccDNA formation.

While some biological functions and disease states including gene regulation, drug resistance, signal communication, aging and tumorigenesis are associated with eccDNA,^7^ little is known about how cf‐eccDNA forms. Therefore, a better understanding of how eccDNA is formed may allow us to establish novel diagnostic and therapeutic tools. In line with this notion, Lv et al. presented that there is mostly (66.36%) a direct repeat sequence (4‐ to 18‐bp) near eccDNA junction sites along with a pair of trinucleotide palindromic repeats with 4‐bp “spacers” in between flanking the eccDNA start and end sites.^4^ Although most of the eccDNA contains direct repeat sequences and palindromic repeats, pointing a possible involvement of homologous recombination‐mediated circularization and microhomology‐mediated end joining process after DNA breaks and contributing to our understanding of eccDNA formation, a large portion of eccDNAs still does not contain these repeats that necessitate further mechanistic work to fully elucidate how/when these circular DNA fragments form.

More interestingly, while the characterization of urinary cf‐eccDNA was similar in terms of size, GC content and motif signature between healthy individuals and patients, samples from patients with advanced CKD (stage 3–5) revealed higher levels of urinary cf‐eccDNA compared to healthy individuals. Although there was a difference in the age parameter between the groups (CKD patients being relatively older), no correlation was found with age and cf‐eccDNA amount, supporting the strong association between eccDNA level and CKD state. Further investigation on characterization of eccDNA from patients demonstrated a frequently detected miRNA eccDNA group in CKD patients. It is quite fascinating that these enriched miRNA‐encoding genes are consistent across patients and are associated with renal and urinary disorders.^8^, ^9^ These findings now require validation through larger cohort clinical studies that include not only CKD patients with advanced stage but also other CKD stages along with other renal and urinary diseases. It would also be interesting to investigate various non‐urinary diseases such as cancer and sickle cell disease, where cfDNA is commonly researched and applied as a diagnostic tool.

These DNA molecules are likely to derive from kidney, urinary tract, as well as plasma. Higher levels of urinary cf‐eccDNA are therefore likely in patients with CDK given higher renal cellular apoptosis, DNA damage and inflammation in these patients. Although many studies reported the presence of cf‐eccDNA in other body fluids, this study proposes one possible mechanism on how these circular DNAs are cleared from the body. As the method used in the study cannot differentiate eccDNA from plasma or urinary tract, this observation and proposed route of clearance for plasma eccDNA should be validated in future studies.

Lv et al. provides a compelling study reporting on the first report of the presence of urinary eccDNA and proposes a potential diagnostic method for CKD. These results are promising for a non‐invasive, straightforward biomarker assay development for not only CDK but probably for all other apoptotic diseases (particularly renal and urinary diseases). As it was previously reported that linear cf‐DNAs in urine samples are highly degraded,^10^ and varying cf‐eccDNA profiles among patients were demonstrated in this study, further studies are highly warranted to show stability/reliability of circular DNA in urine and applicability of the detection method as a diagnostic tool. These studies should be extended to patients with early stage states to explore potentials of disease recurrence prevention, early detection, risk stratification, optimal therapy selection and treatment success/response monitoring.

## CONFLICT OF INTEREST

The author declares that there is no potential conflict of interest.
